# Telomere Length Affects the Frequency and Mechanism of Antigenic Variation in *Trypanosoma brucei*

**DOI:** 10.1371/journal.ppat.1002900

**Published:** 2012-08-30

**Authors:** Galadriel A. Hovel-Miner, Catharine E. Boothroyd, Monica Mugnier, Oliver Dreesen, George A. M. Cross, F. Nina Papavasiliou

**Affiliations:** 1 Laboratory of Lymphocyte Biology, The Rockefeller University, New York, New York, United States of America; 2 Institute of Medical Biology, Immunos, Singapore; 3 Laboratory of Molecular Parasitology, The Rockefeller University, New York, New York, United States of America; University of California, Los Angeles, United States of America

## Abstract

*Trypanosoma brucei* is a master of antigenic variation and immune response evasion. Utilizing a genomic repertoire of more than 1000 Variant Surface Glycoprotein-encoding genes (*VSGs*), *T. brucei* can change its protein coat by “switching” from the expression of one *VSG* to another. Each active *VSG* is monoallelically expressed from only one of approximately 15 subtelomeric sites. Switching *VSG* expression occurs by three predominant mechanisms, arguably the most significant of which is the non-reciprocal exchange of *VSG* containing DNA by duplicative gene conversion (GC). How *T. brucei* orchestrates its complex switching mechanisms remains to be elucidated. Recent work has demonstrated that an exogenous DNA break in the active site could initiate a GC based switch, yet the source of the switch-initiating DNA lesion under natural conditions is still unknown. Here we investigated the hypothesis that telomere length directly affects *VSG* switching. We demonstrate that telomerase deficient strains with short telomeres switch more frequently than genetically identical strains with long telomeres and that, when the telomere is short, switching preferentially occurs by GC. Our data supports the hypothesis that a short telomere at the active *VSG* expression site results in an increase in subtelomeric DNA breaks, which can initiate GC based switching. In addition to their significance for *T. brucei* and telomere biology, the findings presented here have implications for the many diverse pathogens that organize their antigenic genes in subtelomeric regions.

## Author Summary

A broad array of human pathogens (including bacteria, fungi and parasites) vary the proteins on their cell surface to escape the immune response of their hosts. This process, called antigenic variation, relies on a repertoire of variant protein encoding genes in the genome and the organism's ability to accurately switch from the expression of one variant gene to another. A common theme in both the diversification of these variant genes and the mechanisms required for their expression is that they are often located near the ends of chromosomes. The ends of chromosomes are protected by structures called telomeres. Regions near the telomere are referred to as subtelomeric and are commonly thought to be comparatively unstable DNA sites. It is therefore intriguing that organisms that rely on antigenic variation for survival would organize their critical survival genes in these sites. *Trypanosoma brucei* is a model organism for the study of antigenic variation. The causative agent of African sleeping sickness, this unicellular parasite possesses an antigenic repertoire of unparalleled diversity, which can only be expressed from specific subtelomeric sites. Here we use the power of the *T. brucei* model to investigate the effect of telomere length on antigenic variation.

## Introduction

*Trypanosoma brucei* is an extracellular human pathogen with an unparalleled capacity to evade host humoral immunity. The causative agent of African sleeping sickness in humans and nagana in cattle, *T. brucei* is transmitted into the bloodstream of its host by a tsetse vector and can grow to densities as high as 10^9^ cells per milliliter of blood. The parasitemia is cyclically diminished to nearly undetectable levels, to be followed by another wave of immense growth [1]. These rounds of parasitemia reflect the battle between the host immune system and the pathogen's elegant mechanisms of immune evasion.

At the forefront of this battle is the *T. brucei* cell surface, which is primarily composed of about 10^7^ copies of a single, densely packed Variant Surface Glycoprotein (VSG) [2]. VSG is highly immunogenic, yet *T. brucei* escapes immune recognition by switching the monoallelic expression of one *VSG*-encoding gene to another [1], [3]. This process of surface antigen variation is made possible by a genomic repertoire of more than 1000 highly divergent *VSG*-encoding genes and pseudogenes [4], [5], [6]. Furthermore, existing *VSGs* can recombine to form novel mosaic *VSGs*, making the depth of the repertoire potentially limitless [7].

*VSGs* are encoded throughout the *T. brucei* genome, which consists of 11 megabase chromosomes, numerous intermediate chromosomes, and ∼100 minichromosomes [6], [8]. Although most *VSGs* are found in *VSG* arrays within megabase chromosomes or singly on minichromosomes, they can only be transcribed by bloodstream-form parasites from one of the ∼15 Bloodstream Expression Sites (BES) at a time. Proper expression of VSG on the cell surface is essential for survival [1]. Each BES is composed of a collection of Expression Site Associated Genes (*ESAGs*), a long repetitive element (70-bp repeats), and a terminal *VSG* encoding gene, which are transcribed from one upstream promoter and spliced into separate mRNAs before translation [4], [9]. All known BESs are located within 60 kb from the end of the chromosome positioning the expressed *VSG* within >2 kb of repetitive telomeric DNA [4]. The organization of surface antigen encoding genes in subtelomeric regions is a common theme among pathogens that employ antigenic variation to evade host defenses [10], [11].

There are three predominant mechanisms by which *T. brucei VSG* switching can occur: In Situ (IS) transcriptional *VSG* switching — the inactivation of one BES coupled with activation of transcription from a new BES [12], [13], [14], [15], Reciprocal Telomeric Exchange (TE) — a homologous recombination event between two chromosome ends resulting in the balanced transfer of a new *VSG* to the active BES and the previously active *VSG* to a silent BES [16], [17], and Duplicative Gene Conversion (GC) — a non-reciprocal transfer of a *VSG* containing DNA to the active BES that results in loss of the previously active *VSG* from the genome [18], [19], [20], [21]. GC is predicted to account for the majority of *VSG* switching under natural conditions because it is the only mechanism that permits the expression of non-telomerically-encoded *VSG* genes. How each of these mechanisms is orchestrated is largely unknown.

Although the mechanistic basis of each type of *VSG* switch is unknown, it had long been predicted that GC would be initiated by a DNA break. Recent studies have shown that the artificial induction of a DNA double-stranded break (DSB) proximal to the BES repetitive region upstream of the active *VSG* increases the frequency of *VSG* switching by as much as 250-fold, recapitulating the rate estimated in natural isolates [22], [23]. As predicted, *VSG* switching under these conditions occurred mainly by GC [22]. Furthermore it was shown that DNA breaks accumulate in the repetitive region of the active BES [22]. However, the natural source of DNA breaks that precipitate GC remains a mystery.

A proposed source of GC initiating DSBs is related to the proximity of the BES encoded *VSGs* to the telomere (usually within >2 Kb) [4], [24]. The actively transcribed BES frequently experiences large stochastic terminal deletions, which are hypothesized to result from the very high levels of transcription at the end of the chromosome [25], [26]. Thus it has been suggested that when the telomere of the chromosome harboring the active BES is short the DNA breaks precipitating telomeric deletions would occur upstream of the *VSG*, resulting in an antigenic switch [24]. This claim was further correlated to the fact that strains of *T. brucei* that have been recently isolated from nature, who switch at a rate of approximately 10^−2^–10^−3^, have shorter telomeres than laboratory-adapted strains, whose rate of switching can be are 100–10,000-times lower [27], [28], [29], [30]. In all, this suggested an inverse correlation between telomere length and the amount antigenic switching in *T. brucei* [24], yet there were no data in direct support of this hypothesis.

*T. brucei* strains with the protein component of telomerase deleted *(TERT^−/−^)* are unable to repair telomeric breaks (such as those that occur frequently at the actively transcribed BES) and undergo progressive shortening of all the telomeres in the genome by 3–6 bp/Population Doubling (PD) [31]. Previously it was shown that when the telomere of a *TERT^−/−^* isolate is short, the actively expressed *VSG* is lost over the course of several weeks and replaced by a new *VSG* gene, which suggested an increase in *VSG* switching [32]. However, because of the time span in which those experiments took place, that study could not differentiate between the equivalent possibilities of an increase in switching versus the death of short-telomere clones being replaced by a subpopulation of switchers arising at the normal *in vitro* frequency [32].

In this study we directly tested the proposed correlation between *VSG* switching and telomere length. Using updated techniques, we compared the frequency of *VSG* switching between strains with wild-type length or shortened telomeres at the active BES. Large populations of switched clones were analyzed to identify their mechanism of switching and determine if those with short telomeres switch by way of a preferred mechanism. The findings presented here provide experimental support for the hypothesis that telomere length directly affects the frequency of *VSG* switching, and answer long-standing questions about the relationship between *VSG* switching, telomere length, and gene conversion.

## Results

### Telomere length affects antigenic switching frequency *in vitro*

**Figure 1:**
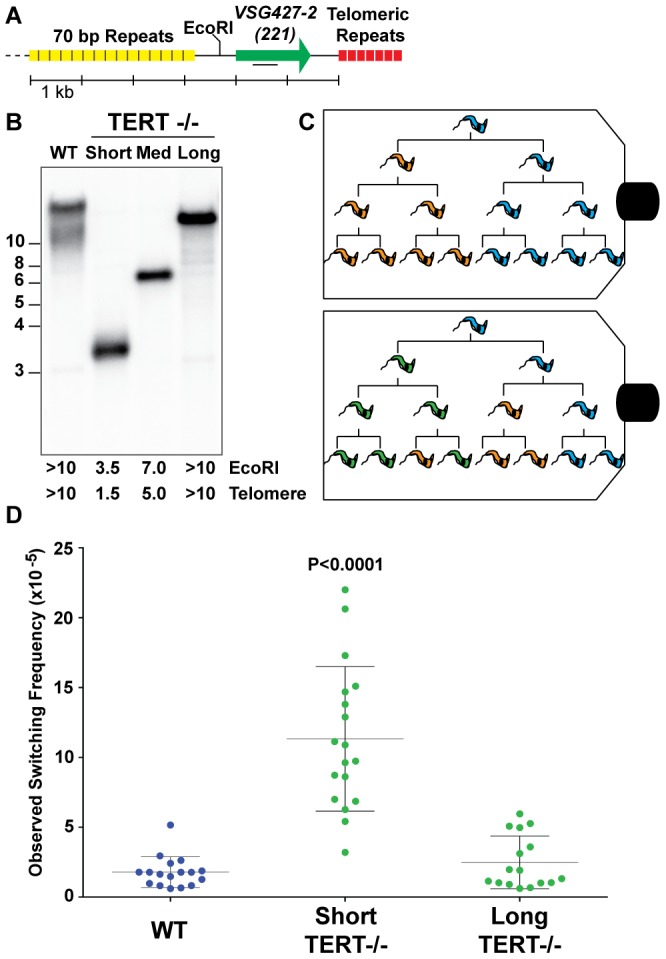
Correlating telomere length and *VSG* switching. (A) Map displays proximity of the active *VSG* to the telomere, the position of the EcoRI site, and the position of DNA probe used in Southern blotting. (B) Lengths of wild-type (WT) and *TERT^−/−^* mutant telomeres shown by Southern blot analysis probed with *VSG427-2 (221*). (C) Illustration of two possible outcomes of fluctuation analysis resulting from a single trypanosome cell. (D) Observed Switching Frequency (OSF) of WT (n = 17), *TERT^−/−^* Short (n = 18), and *TERT^−/−^* Long telomere (n = 16) strains are shown. Each dot represents the OSF value arising from a single cell clone following fluctuation analysis for an equivalent number of population doublings. The P value applies to the comparison of *TERT^−/−^* short-telomere to either WT or *TERT^−/−^* long-telomere data sets.

To address the effect of telomere length on *T. brucei* antigenic switching, we first isolated strains with various telomere lengths at their active BES (BES1 expressing *VSG427-2 [221]*). The telomere of BES1 in a population wild-type isolates (WT) can range from ∼10–15 kb (FIG. 1. A). *TERT^−/−^* clones with short (∼1.5 kb), medium (∼5.0 kb), and long (>10 kb) BES1 telomeres were isolated and characterized by Southern blot (FIG. 1. B). The active BES telomere in a *TERT^−/−^* strain is not only prone to progressive shortening (3–6 bp/PD) but also massive truncations [31]. Thus, medium- and long-telomere clones can only be handled for a minimal number of passages before they shorten (as evidenced by the smear under the primary band in FIG. 1. B – “Long”). In contrast, critically short telomeres are stabilized by an unknown, telomerase-independent, mechanism that appears to be unique to *T. brucei* [33]. Thus resulting in the short-telomere clone used here (FIG. 1. B – “Short”), which can be stably maintained at a length of ∼1.5 kb for numerous passages.

Populations of trypanosomes, as with any organism, are heterogeneous and this can affect both the expressed *VSG* and telomere length. Furthermore, accurate determination of the frequency of *VSG* switching requires that the populations being compared undergo a comparable number of population doublings (PD) during the experiment. Therefore, WT, *TERT^−/−^* short- and long-telomere clones with similar growth rates (FIG. S1) were grown from single-cells to ∼5×10^7^ *in vitro*, thereby performing a modified Luria-Delbrück fluctuation analysis [34] (FIG. 1C). The *VSG* switching frequency of the resulting populations of trypanosomes was determined using the previously published magnetic-activated cell sorting (MACS) depletion of the initiating *VSG* followed by flow cytometry quantification [22].

*TERT^−/−^* short clone populations switched their expressed *VSG* at a significantly (P<0.0001) higher frequency (11.3×10^−5^±4.6) than both WT (1.8×10^−5^±1.1) and *TERT^−/−^* long-telomere clones (2.5×10^−5^±1.9), which were not significantly different from each other (P = 0.3136) (FIG. 1D). The *TERT^−/−^* long-telomere clones serve as a proxy for *TERT* complementation in this study because a previous study showed that ectopic *TERT* expression results in rapid elongation of the active site telomere (∼160 bp/PD) [33], which prevents the analysis of a short-telomere *TERT* complemented clone by this method. The switching frequencies of the *TERT^−/−^* short-telomere clones covered a broad range of values (3.2–22×10^−5^), which correspond to a 6- to 36-fold increase in switching compared to WT. These data might be explained by a stochastic increase in subtelomeric DNA breakage that occurs when the telomere of the active site is short. Alternatively, in the absence of *TERT* complementation data, the increase in switching could arise from a combinatorial effect of having a short telomere in the context of a telomerase mutant. In either case these data support the notion that subtelomeric breaks promote antigenic switching.

The output of the *VSG* switching assay represents the proportion of the input population that is no longer expressing the starting *VSG* type, but this value does not account for replication of progeny, the number of *VSG* switching events intervening before the final measurement, or potential genotypic diversity of phenotypically identical cells in the resulting population (FIG. 1C). We therefore adopted the nomenclature Observed Switching Frequency (henceforth referred to as switching frequency or OSF) to indicate the limitations of this measured value (FIG. 1D).

### Expressed *VSG* diversity following *TERT^−/−^* short-telomere switching

**Figure 2:**
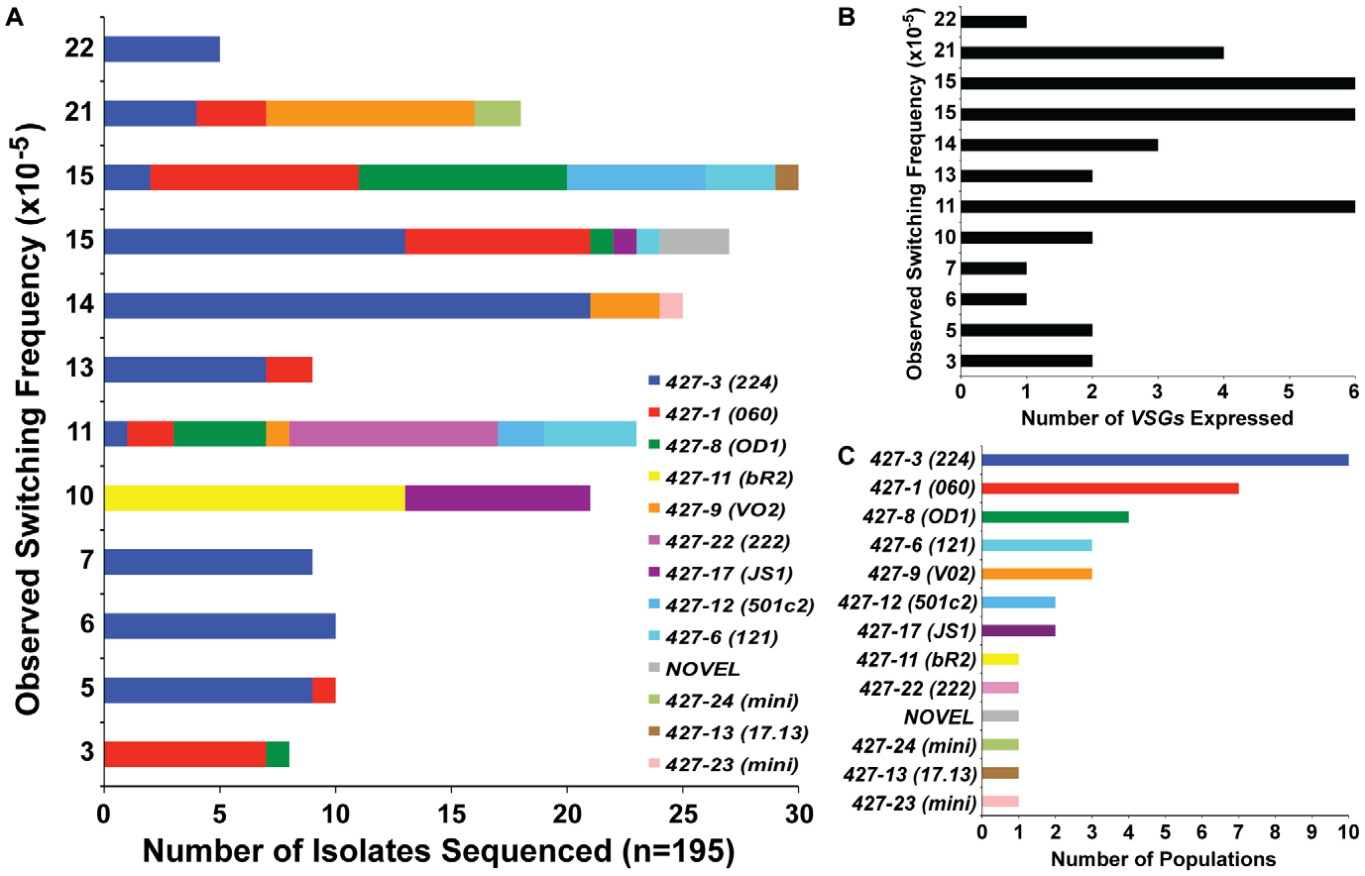
*VSGs* expressed in *TERT^−/−^* short-telomere switched populations. (A) Depiction of the diversity of *VSGs* present in specific populations of *TERT^−/−^* short-telomere switchers. X-axis shows the number of times each *VSG* was identified by bacterial colony sequencing versus the OSF value (same as those shown in FIG. 1D) on the y-axis. (B) Correlation of the total number of *VSGs* expressed in each population (x-axis) versus the OSF of that population (y-axis). (C) The number of populations (out of 12 total) (x-axis) in which each expressed *VSG* (y-axis) was identified.

Hypothetically, populations that switch more often will contain a greater diversity of expressed *VSGs*. Thus we predicted that populations with higher OSF values would contain a larger diversity of *VSG* transcripts. To investigate this prediction, the MACS eluates from 12 of the 18 *TERT^−/−^* short-telomere switched populations (FIG. 1D) were grown to a sufficient extent for RNA extraction to identify the expressed *VSGs*, which were then compared with the determined OSF for that population (FIG. 2A, y-axis values correspond directly with FIG. 1D *TERT^−/−^* short data). This type of analysis is only possible due to the extensive nucleotide sequence diversity of *VSG*-encoding genes, which allows each *VSG* to be accurately distinguished from another [35]. Although there was a subtle trend of populations with OSF>10 to express a greater diversity of *VSGs* (∼3–6) than those with an OSF<10 (1–2 *VSGs*) (FIG. 2B), we did not observe a linear relationship between the switching frequency of these populations and the depth of their *VSG* diversity (FIG. 2). This is highlighted by the fact that the highest OSF (22×10^−5^) of this set contained only one expressed *VSG* (*VSG427-3 [224]*) (FIG. 2A & 2B).

**Figure 3:**
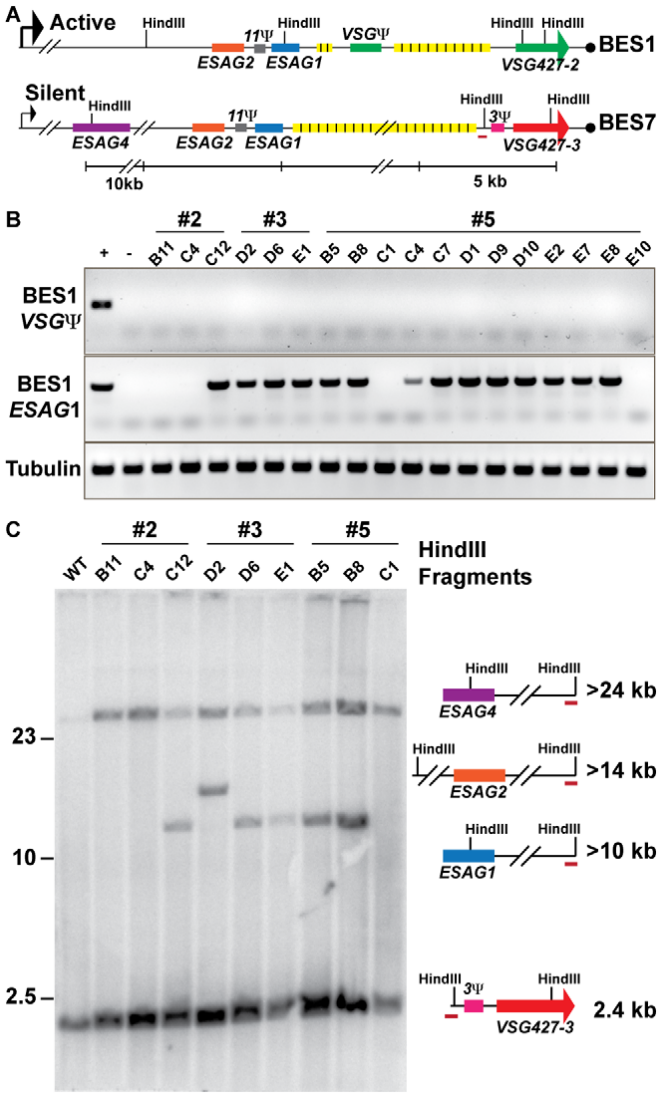
Genetic diversity of *VSG427-3 (224)* switchers. (A) Comparative maps of BES1 (active) and BES7 (silent; source of *VSG427-3*) with critical experimental features illustrated: HindIII sites, genes (*ESAG1 & VSG Ψ*), and BES7-unique probe (red line). (B) PCR products from 18 *VSG427-3* switcher originating from 3 *VSG427-2* starting clones (#2, #3, & #5) are shown for BES1 *VSG Ψ*, BES1 *ESAG1*, and Tubulin control in comparison to positive (WT) and negative (BES1 deletion strain courtesy of Kim & Cross). The control strain demonstrates the presence or absence of features in the genome. (C) FIGE Southern blot analysis of *VSG427-3* switchers from HindIII digested gDNA probed with BES7-unique probe (BES7 red underline). Illustrations and predicted sizes of restriction fragments shown to the right of the blot image.

It is worth noting that the diversity of *VSGs* identified in *TERT^−/−^* short-telomere *VSG* switching assays was similar to those seen in other studies [1]. *VSG427-3 (224)* was the most commonly observed *VSG*, which was present in 10 of the 12 populations analyzed, and the sole *VSG* expressed in 3 of those populations (FIG. 2A & 2C). The second most commonly expressed was *VSG427-1 (060)* in 7 populations, followed by *VSG427-8 (OD1)* in 4 populations (FIG. 2C). These data support previous *in vivo* and *in vitro* studies suggesting that certain *VSGs* are favored donors and that *VSG* switching follows a semi-predictable order [36]. In addition to the predictably expressed *VSGs*, we isolated two *VSGs* that were recently demonstrated to reside on minichromosomes (*427-23 & 427-24*) [22] and a *VSG* that had not previously been annotated (FIG. 3, “NOVEL”).

### Phenotypic diversity under-represents genetic diversity of switched isolates

The lack of a clear linear relationship between the switching frequency and the depth *VSG* diversity (FIG. 2) could arise from two alternative biological situations: extensive propagation of an early switch event (FIG. 1C, top) or multiple switch events that result in expression of the same *VSG* (FIG. 1C, bottom). As noted, the population of *TERT^−/−^* short telomere with the highest OSF (22×10^−5^) contained only *VSG427-3 (224)* (FIG. 2A). Did this population arise from one or multiple *VSG* switch events? To address this question, we isolated *TERT^−/−^* short-telomere trypanosomes that had switched from *VSG427-2* (expressed from BES1) to *VSG427-3* (originating from BES7) from three separate fluctuation analysis experiments. The genotypic differences among isolates from the same starting clone were then compared by PCR and Southern blot analysis (FIG. 3).

Eighteen switchers expressing *VSG427-3* were isolated from three populations (3 clones from two populations [#2 & #3] and 12 from the third [#5]). All 18 clones had switched to *VSG427-3* by GC (expressed from BES1), as evidenced by the loss of the *VSG pseudogene* from BES1 (FIG. 3A; BES1 map *VSGΨ* green box). Using PCR primers unique to *ESAG1* [4] in BES1, we determined that *VSG427-3* switchers from populations #2 and #5 contained a mixture of clones that had either lost or retained BES1 *ESAG1* during GC (2 of 3 in population 2 and 2 of 12 in population 5 had lost *ESAG1*). Loss of BES1 *ESAG1* indicates that resolution of GC occurred upstream of *ESAG1*. Therefore, clones expressing *VSG427-3* in these populations arose from at least two distinct *VSG* switching events.

To further analyze the genotypes of the *VSG427-3* switchers, we used a unique region in BES7 to probe a HindIII-digested large-fragment separation gel and Southern blot (FIG. 3A). There are two HindIII sites in BES1 between the repeat region and *ESAG4* that distinguish it from BES7 (FIG. 3A). Thus, GC resolution downstream of *ESAG1* results in the formation of a >10 kb fragment, upstream of *ESAG1* but downstram of *ESAG2* results in a >14 kb fragment, and upstream of *ESAG2* but downstream of *ESAG4* results in a fragment >24 kb (FIG. 3C). Because BES7 is intact in the genome regardless of switching, all strains produce the >24 kb and ∼2.4 kb fragments. Exact prediction of product sizes for fragments resolved beyond the BES7 repeat region (FIG. 3A, yellow boxes) is not possible due to missing sequence data [4].

Southern blot data largely agreed with PCR data (FIG. 3B), such that the *VSG427-3* switchers that retained BES1 *ESAG1* by PCR produced the restriction fragment associated with resection downstream of *ESAG1* (>10 kb fragment present for #2 = 2/3, #3 = 2/3, & #5 = 3/12 [only partial data shown]). However, one isolate from clone 3 retained *ESAG1* but produced a fragment that was predicted to result in the loss of *ESAG1* (#3 D2, >14 kb). This combination of data could arise from a recombination event of unpredicted complexity. These results further demonstrate that each population of phenotypically identical *VSG427-3* switchers analyzed are composed of at least 2 genotypes, and therefore must arise from multiple genetic events. Fine mapping or sequencing the point of resection for these strains could show further switching diversity, but is not possible due to the >90% sequence similarity between BES1 and BES7 [4]. Therefore, the OSF values displayed in figure 1 under-represent genetic switching frequency by not accounting for the number of *VSGs* expressed in the population (FIG. 2) and the genetic diversity of the switched population (FIG. 3).

### *TERT^−/−^* short-telomere clones preferentially switch by duplicative gene conversion

**Figure 4:**
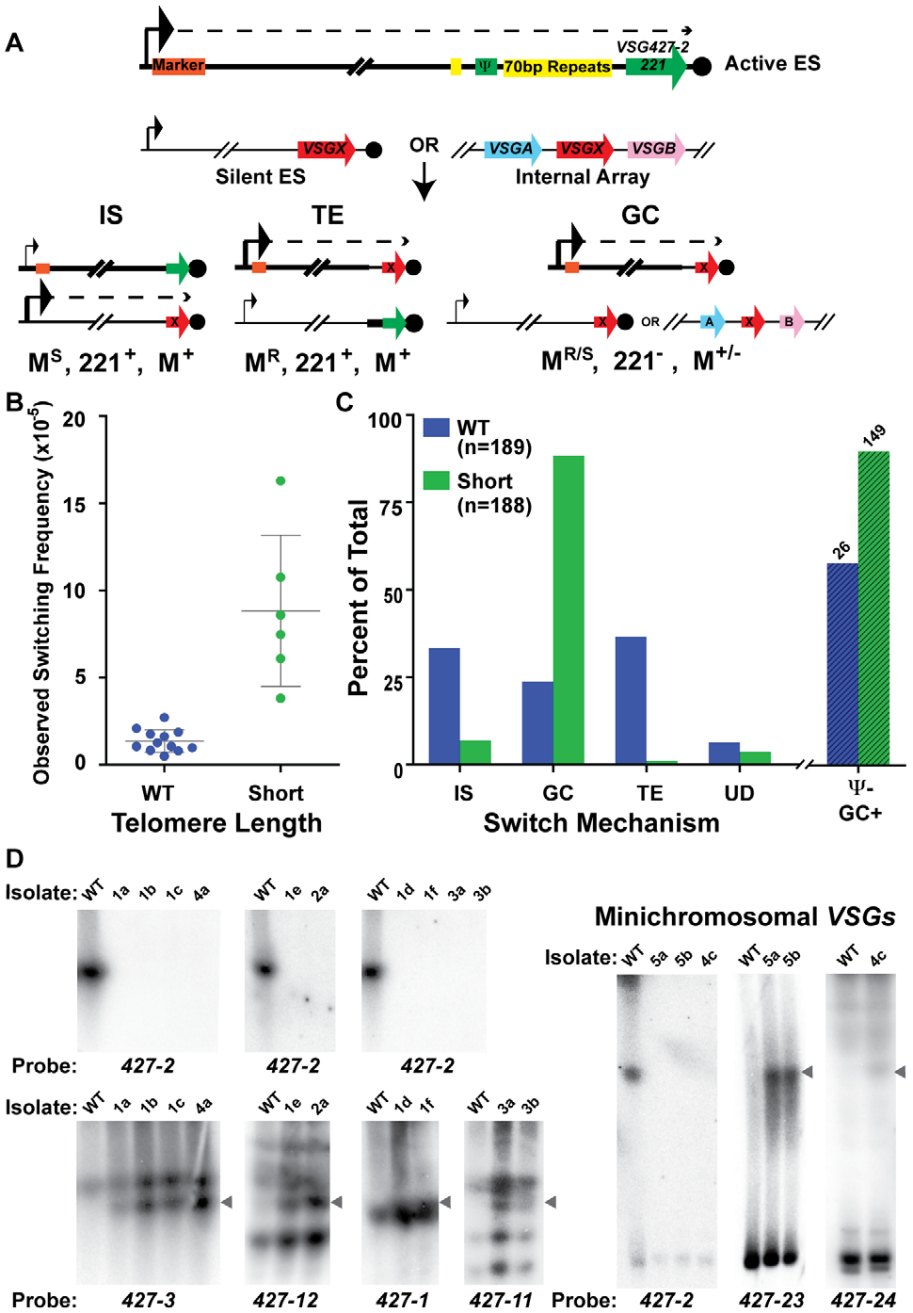
Mechanisms of *VSG* switching in WT and short-telomere isolates. (A) Cartoon depicting the critical details of the three predominant mechanisms of *VSG* switching- IS (In situ), TE (telomere exchange), and GC (duplicative gene conversion). Large arrowhead indicates active promoter; small arrowhead depicts the silent promoter; dashed line indicates transcription; orange box shows the position of the antibiotic selection marker. *VSG427-2* nomenclature is used interchangeably with its colloquial name *VSG221* (B) OSF of the populations of WT (blue, n = 12 populations) and *TERT^−/−^* short-telomere (green, n = 6 populations) switchers. (C) Percent of WT (blue, n = 189) and *TERT^−/−^* short-telomere (green, n = 188) *VSG* switched isolates versus switching mechanism: IS (in situ), GC (all gene conversion = GC+ES−GC), TE (telomere exchange), or UD (undetermined). Percent loss of BES1 *VSG* pseudogene from GC (“Ψ− GC+”) switched isolates (WT n = 26 & Short n = 149) is shown beyond the broken line (WT, lined blue bar & short-telomere, lined green bar). (D) RAGE separated chromosomes blotted and probed with *VSG* specific probes for a subset of *TERT^−/−^* short-telomere switched clones.

To determine if telomere length affects the mechanism of switching as well as frequency, populations arising from single cells clones of wild-type and *TERT^−/−^* short-telomere strains were MACs sorted, their OSF determined (FIG. 4B – using the same methodology used to produced the data in FIG. 1 with new starting populations), and plated to limiting dilution. The secondary clones were then analyzed for the expression of *VSG427-2* (*221*) by high-throughput screening (HTS) flow cytometry, and all cultures not expressing *VSG427-2* were deemed “switched clones.” From 12 populations of WT, we screened 1617 secondary clones, identifying 189 WT switched clones (12% of the post-MACS screened population), whereas screening of 255 secondary clones from 6 populations of *TERT^−/−^* short-telomere clones resulted in a similar number (188) of switched clones to be further analyzed (74% of the post-MACS screened population). The mechanism of switching for all secondary clones was then determined, based on three criteria: (1) resistance or sensitivity to a BES1 promoter-proximal antibiotic marker (WT marked with hygromycin [HYG] & *TERT^−/−^* marked with blasticidin [BSD]), (2) the presence or absence of *VSG427-2 (221)* in the genome, and (3) the presence or absence of the promoter-proximal resistance marker in the genome. Thus the switch type can be counted in the following way: IS events correspond to Marker^S^, *427-2(221)* ^+^, Marker^+^; TE events are Marker^R^, *427-2(221)* ^+^, Marker^+^; GC results in Marker^R^, *427-2(221)* ^−^, Marker^+^; ES GC (Expression Site Gene Conversion), a subtype of GC in which the entire active BES is replaced by the donor BES, are Marker^S^, *427-2(221)* ^−^, Marker^−^; and UD, for Undetermined, when switched clones did not fit the other criteria (FIG. 4A & TABLE S3). These data were also used to establish a minimal number of independent switch events. By creating a matrix based on the 12 known combinations of phenotype and genotype analyzed (*VSG427-3 [224]* expression as determined flow cytometry [data not shown]), switch type [IS, TE, GC, ES GC or UD], & BES1 *VSG* Ψ^+/−^) against the source populations (12 for WT & 6 for Short), we determined that the 189 WT switched clones arose from at least 47 independent switchers and the 188 Short switched clones from at least 25 (TABLE S3). The higher number for WT is an artifact of the increased complexity of its matrix (i.e. more originating populations). Determining the mechanism of switching in *TERT^−/−^* long-telomere clones was not possible because of their propensity to break at the active BES, which results in the rapid formation of a heterogenic population of telomere lengths.

WT *VSG* switched clones produced similar average levels of IS (33%), GC (24%), and TE (37%). This was in contrast to the *TERT^−/−^* short-telomere *VSG* switchers, which showed a clear preference for GC-based switching (88%), only a small amount of IS (7%) and negligible TE (1%) (FIG. 4C [“GC” shown is the sum of both GC and ES GC] & TABLE S1, S2, S3). Switching by GC for a subset of *TERT^−/−^* short-telomere clones was further confirmed by pulsed-field gel separation of chromosomes followed by Southern blot analysis using specific *VSG* probes, which showed both the loss of the initiating *VSG* (*427-2*) and the duplication of the newly expressed *VSG* to the active BES (FIG. 4D). These data, in conjunction with published data that induction of a DNA break in the active site initiates a GC based switch [22], suggest that a short telomere at the active site can increase switch initiating subtelomeric DNA breaks. Further mathematical analysis of switched clone data suggested that the heightened level of GC could account for the overall increase in switching frequency observed when the telomere is short (mathematical validation shown in Experimental Procedures & TABLE S3). The same analytical process showed that the frequency of IS switching (**F**_IS_ = average OSF×% IS, for both WT and *TERT^−/−^* short-telomere clones) was not significantly different between WT and *TERT^−/−^* short-telomere clones (TABLE S3). To further analyze the nature of the GC events in WT and *TERT^−/−^* short, all isolates identified as GC were assayed for the presence of BES1 *VSG* pseudogene (*PSD* or Ψ) in the genome, a parameter that indicates the location of GC resolution. BES1 *VSG PSD* was lost from *TERT^−/−^* short-telomere switchers (90%) ∼30% more often than WT (58%) (FIG. 4C, lined bars “Ψ− GC+”). This indicates that the resolution of GC events in *TERT^−/−^* switchers often occurs farther upstream than in WT *VSG* switchers and suggests that the initiating lesion itself may occur farther upstream when the telomere is short.

The body of isolated switcher data was further analyzed to determine if individual populations, OSF values, or isolation dates, affected the propensity to switch by a given mechanism (TABLE S3, FIG. S2 & S3). Each initiating clone results in a population of secondary switched clones with a somewhat variable percentage of each switching mechanism. Generally, population variations did not affect the results, but there were two WT populations (#2-5 & #2-6) with a large number of switchers (34 & 25, respectively) that had a high percentage of TE (76% & 80%, respectively) (FIG. S2 & TABLE S3), which gave the average WT TE value of 37%. If reanalyzed, the median values for WT were IS = 37%, GC = 20%, and TE = 19%, thus reducing the overall significance of TE. Analysis of the percent switch mechanism in comparison with the switching frequency for each population showed a very subtle trend for populations with higher OSF values to contain a higher level of diversity in the mechanisms represented, which was similarly true for both WT and short-telomere isolates (TABLE S3). Due to different growth rates following limiting dilution, *VSG* switch isolates were selected 7, 9, or 11 days after initial plating. Analysis of the *VSG* switch mechanism by isolation date for WT and *TERT^−/−^* short-telomere clones displayed a trend of increased IS and decreased GC with time (this pattern was more apparent in WT), while TE did not (FIG. S3). This suggests that isolates that switch by IS initially grow more slowly than those that switch by GC; the basis of this difference is unknown. None of the alternative analysis of the data in this section detract from the central result, namely that *TERT^−/−^* short-telomere clones switch preferentially by GC.

## Discussion

Telomeres are structures of DNA and protein that protect the ends of chromosomes from DNA loss and damage. Yet, somewhat counterintuitively, *T. brucei* is only one of the many pathogens whose critical antigenic diversity genes are organized in potentially fragile subtelomeric regions [10], [37]. In 2007 Dreesen et al. proposed a model in which telomere length (at the active BES) had an inverse correlation with the frequency of *VSG* switching in *T. brucei*. The model also predicted that switching at short telomeres would occur by GC, resulting from an increase in BES internal DSBs when the telomere is short [24]. The 2007 proposal was based on a correlation between two main bodies of data: 1) the telomeres of laboratory-adapted strains are longer than those of strains recently isolated from nature [30] & 2) populations of *TERT^−/−^* short-telomere strains progressively loose the initially expressed *VSG*, which suggested, but did not demonstrate an increase in the frequency of *VSG* switching [33]. Although this model gained popular support, its predictions were unsubstantiated.

Here we have used updated techniques to rigorously test the proposed correlation between telomere length and *VSG* switching. Our data demonstrate, for the first time, that antigenic switching in *T. brucei* increases in a *TERT^−/−^* mutant when the telomere of the active BES is short (in direct contrast to *TERT^−/−^* strains with long a telomere whose switching is like WT) (FIG. 1). Furthermore, we show that the increase in switching can be accounted for by a significant increase in GC (in comparison with other measured switching mechanisms), which is likely due to an increase in DNA breaks in the region upstream of the *VSG* in the active/short-telomere BES (evidenced by an increased loss of *VSG* pseudogene [Ψ]) (FIG. 4).

There are two equally valid mechanistic interpretations of these data. The first is that telomere shortening *per se* leads to an increase in the frequency of DNA breakage in locations that surround the *VSG*, thus precipitating a switching event, as hypothesized originally by Cross and colleagues [24]. Alternatively, the phenotype we observe might be due to a telomere-capping defect, which would be the result of the combined effect of shortened telomeres in the context of a telomerase-null mutant. Indeed, such a capping defect has been shown to increase gene conversion in the budding yeast *Kluyveromyces lactis* [38]. To distinguish between these two mechanistic possibilities, we have made concerted efforts to complement telomerase expression in *TERT^−/−^* short-telomere *T. brucei* so that their switching frequency could be compared to un-complemented *TERT^−/−^* short-telomere strains. However, we have been unable to set up a strictly regulated inducible system to reconstitute *TERT* expression *while retaining a short telomere at the active expression site* (even at exceedingly low expression levels). This is due to the inherent leakiness of inducible systems available for *T. brucei*, together with the impressive efficiency of catalytically active telomerase to rapidly elongate a short telomere at the active expression site [30], [31]. Also, the impact of telomerase mutations, if any, on t-loop formation and telomere capping in *T. brucei* is not known at this time. Thus, at the moment, we cannot distinguish between the possibility that the phenotype we observe in the *TERT^−/−^* short-telomere strains is due to a direct effect of telomere shortening, as originally proposed, or a capping defect (combined with telomere shortening) due to lack of telomerase function, along the lines of *K. lactis* [38]. Nevertheless, the general appropriation of conserved mechanisms of chromosome end protection (breakage, lengthening and capping) for the purposes of increased antigenic variation is an intriguing possibility as an aspect of antigenic variation in *T. brucei*.

*T. brucei* strains that have been recently isolated from nature are distinct from their laboratory propagated cousins in that they switch more frequently (approximately 10^−2^–10^−3^) [27], have shorter telomeres [30], and preferentially switch by GC [39]. Thus, it would appear that by producing a *T. brucei* strain with an artificially shortened telomere we have, at least partially, recapitulated these characteristics of a natural isolate. The 10- to 100-fold increase OSF measured in the *TERT^−/−^* short-telomere strain does not cover the approximately 100- to 10,000-fold difference in the rate of switching published between recent isolates (approximately 10^−2^–10^−3^) and laboratory-adapted strains *in vitro* (approximately 10^−5^–10^−6^) [28], [29]. Comparative analysis of switch rate approximations has been a central challenge in the field due to significant variability among the methodologies used and the very small sets of *VSG* switchers (often less than 10) from which these rate approximations were often derived [27], [28], [29]. The OSF value is simply a measurement of the proportion of cells in the population that are no longer coated in the parental *VSG*, which does not account for the phenotypic diversity of *VSGs* in the population (which was included in previous approximate rate derivations) (considered in FIG. 2) or the genotypic diversity (for which no method has ever accounted) (considered in FIG. 3). The findings presented here suggest that the shorter telomeres of natural isolates could contribute to their comparatively high level of *VSG* switching.

The question that persists is, how naturally occurring populations keep their telomeres short? In addition, *T. brucei* telomeres grow by 6–8 bp/PD during growth under laboratory conditions (a process that has only been observed in this organism) [25], [30]. What is distinct between the natural *T. brucei* lifecycle and their laboratory propagation that could account for the observed differences in telomere lengths? Although telomerase regulation in *T. brucei* is not understood, in other organisms telomerase is activity is regulated by the cell cycle such that telomeres only lengthen in the transition from S phase to G2 [40], [41], [42]. If this were also the case for trypanosomes, the organisms increased *in vivo* growth rate (which is more than 2 times higher than *in vitro*) could result in a reduced duration of telomerase activity and thus progressively shorter telomeres. An alternative possibility is that a regulated component of telomere structure or stability, or possibly telomerase itself, is affected when trypanosomes are not permitted to undergo their natural life cycle, which includes passage through the tsetse. In support of this hypothesis, laboratory adapted *T. brucei* can recover its *in vivo* switch rate following passage through the tsetse [29]. Although little is known about the regulation of telomerase and other telomere-associated proteins in trypanosomes, perhaps this is a missing connection between the switching behavior of natural isolates and extensively adapted *T. brucei* strains.

## Materials and Methods

### *Trypanosoma brucei*

Cell lines were generated from Lister427 bloodstream-form trypanosomes derived from the ‘single marker’ line [43], with a hygromycin resistance marker at the BES1 promoter [22] (“wild-type” [WT]), or homozygous telomerase (Gene ID: 3664223 & protein accession: XP_829083) deletion mutant with blasticidin resistance marker at the BES1 promoter (*TERT^−/−^*) [31], [32]. *TERT^−/−^* short and long active-site telomere BES clones were isolated from single cell cultures of *TERT^−/−^* and telomere lengths were determined by Southern Blot analysis (below) [31]. Trypanosomes were cultured *in vitro* in HMI-9 medium at 37°C [44].

### Southern blot analysis

DNA restriction fragments were separated by standard agarose gel electrophoresis (1–15 kb), Field Inversion Gel Electrophoresis (FIGE) [45] (1–25 kb, BIORAD “Program 1”), Rotating Agarose Gel Electrophoresis [46] (“Classical Program” for separation of megabase, intermediate, and minichromosomes), using published methods. Southern blots were produced using established methods of capillary blotting by neutral transfer (GE Scientific). DNA probes were made by PCR amplification using previously published primer sequences [4], [22], ^32^P radiolabeled using Prime-It II Random Labeling Kit (Stratagene), and purified over G-50 microcolumns (GE Healthcare). Blots were probe-hybridized, washed, and visualized by phosphorimaging as described (GE Healthcare).

### OSF determination by MACS and flow cytometry-based fluctuation analysis

Strains expressing *VSG427-2* were single cell cloned by limiting dilution and expanded to a total of ∼5×10^7^ cells prior to MACS isolation and flow cytometry-based quantification of *VSG* switching frequency as previously described [22]. The *VSG* switching frequency, here termed the “Observed Switching Frequency (OSF)”, is a direct measure of the proportion of living (measured by propidium iodide staining) trypanosomes in a population that no longer express *VSG427-2* on their surface, compared to the total input population, both of which are normalized to a control sample (CountBright Beads purchased from and used according to Invitrogen instructions).

### Identification of expressed *VSGs*

Following MACS depletion of *VSG472-2* expressing cells, half of the effluent was removed prior to OSF determination by flow cytomertry and grown in 50 mL HMI-9 with pen-strep until confluent. RNA was extracted, cDNA synthesized, *VSG* RT-PCR amplified and subcloned (pGEM-T Easy, Promega) using established methods (tryps.rockefeller.edu/trypsru2_protocols_index.html, “VSG cloning for mRNA”), prior to sequencing and expressed *VSG* determination by NCBI BLAST.

### Isolation and analysis of *VSG427-3 (224)* switchers

Following MACS depletion of *VSG427-2* expressing cells (from single cell cultures grown for the specific purpose of *VSG427-3* switcher isolation and not OSF determination), the effluent was labeled with anti-224 antibody and bound to a second MACS column. Following standard MACS binding and wash steps the bound trypanosomes (anticipated *VSG427-3* ^+^ switchers) were plunged from the column, cloned and screened for *VSG427-3* expression by flow cytometry. *VSG427-3* expressing clones were grown for genomic DNA isolation [46], which was analyzed by PCR using BES1 *VSG pseudogene* primers (pseudo5-F: 5′- GCGCCGAATTTAATGCAATATGCAACG & pseudo5-R: 5′- GCAGGCCGTCTTTTGAGTTGTAGTAAG) & BES1 *ESAG1* primers (ESAG1 Fw1: 5′-GAGCAAACTGATAGGTTGGAAAAG & ESAG1 Rv1 5′-GCACTGGCGGCCACTCCATTGTC) and HindIII FIGE Southern blot analysis using a BES7 specific probe (FIG. 4A – red bar) produced by PCR using unique primers (BES7 UR1 Fw: 5′-GCAACTAACTACTGTAATTCCC & BES7 UR Rv: 5′-GCTACTAATGTGTTTCAATATGCG).

### Determination of *VSG* switch mechanism

Following expansion of *VSG427-2 (221)* expressing WT and *TERT^−/−^* short-telomere cells from single cells to ∼5×10^7^ total cells and MACS depletion of *VSG472-2* expressing cells as described above [22], the effluent was split into two samples. Half of the resulting cells were used to determine the OSF and the other half was cloned by limiting dilution in 96-well tissue-culture plates. Clone growth was observed for two weeks following initial plating. At 7, 9, and 11 (WT only) days after plating, confluent cultures were split into new 96-well plates and allowed to become confluent. Final identification of *VSG427-2* ^−^ switchers was performed by high-throughput-screening (HTS) flow cytometry using *anti-VSG221* antibody on an LSRII with HTS adaptor for 96 well plate analysis (BD Biosciences). *VSG427-2* negative clones were then analyzed for BES1 promoter activity by antibiotic selection of the BES1 promoter proximal marker (WT: hygromycin & *TERT^−/−^*: blasticidin) and the Southern dot blots for the presence of *VSG427-2 (221)*, the promoter proximal antibiotic resistance gene and BES1 *VSG pseudogene* in the genome (using radiolabeled probes to VSG221, hygromycin, blasticidin, pseudogene). Southern dot blot was adapted for trypanosomes from manufacturers protocols (GE Healthcare) by adding ∼1×10^6^ cells/well to the membrane, cell lysis with denaturation buffer, neutralization, and fixation with UV prior to radiolabeled probe hybridization, washing, and visualization as described above for Southern blot analysis. Primers for PCR production of Southern dot blot probes: 221 Prb Fw (5′- GTAACAGCTACTGCAACAGCGAGC)& 221 Prb Rv(5′- GCTTCTTCAACAAGCTTGGTAACG), HYGRO Prb Fw (5′-GCTCTCGATGAGCTGATGCTTTGG) & HYGRO Prb Rv (5′-GATAGAGTTGGTCAAGACCAATGC), BES1 PSEUDO Fw (5′- CATTAAATTCAAGCGTCTAGACCGCAGC) & BES1 PSUEDO Rv (5′- GCGCGTTGTTCCGTATCTGCTGAGC)

### Mathematical analysis of GC contribution to OSF

If Σ**F**_Short_≈Σ**F**_WT_+Δ**F**_GC_, then GC accounts for the increase in short-telomere OSF. Where **F**_Mech_ = Average OSF×% Mechanism and Δ**F**_GC_ = **F**_GC,Short_−**F**_GC,WT_. First the **F** for each mechanism for both WT and Short were determined, the sum of which is equal to the average OSF for each strain (Σ**F**_WT_ = 1.38 & Σ**F**_Short_ = 8.84). Then the Δ**F**_GC_ was determined and used to show that Σ**F**_WT_+Δ**F**_GC_ = 8.86 which is fundamentally identically to the Σ**F**_Short_ (8.84), thus making Σ**F**_Short_≈Σ**F**_WT_+Δ**F**_GC_ a valid statement (TABLE S3).

## Supporting Information

Figure S1 **Growth of wild-type and telomerase mutant strains.** Comparison of the growth of wild-type (WT) (blue) with *TERT^−/−^* short-telomere (green), and *TERT^−/−^* long-telomere (red) clones.
(JPG)

Figure S2 **Mechanism of switching by clone population.** Comparison of percent switch mechanism (IS: in situ [black], GC: gene conversion [dark blue], ES GC: expression site gene conversion [light blue], TE: telomere exchange [red], UD: undetermined [grey]) by starting clone population. Number of secondary switched clones in each population is shown above the bracket. Wild-type (WT) telomere populations are shown in top graph % short-telomere populations shown in bottom graph.
(JPG)

Figure S3 **Mechanism of switching by clone isolation date.** Comparison of percent switch mechanism (IS: in situ [black], GC: gene conversion [dark blue], ES GC: expression site gene conversion [light blue], TE: telomere exchange [red], UD: undetermined [grey]) by secondary clone isolation day. Number of secondary switched clones in each population is shown above the bracket. Wild-type (WT) telomere populations are shown in top graph and short telomere populations shown in bottom graph.
(JPG)

Table S1 **Switch type determination of WT clones.** Table presents the analysis of the 189 WT single-cell-isolated *VSG* switched secondary clones (supporting data for FIG. 4C – Blue Bars). The table columns from left to right are: isolate identifier (codified name based on originating 96-well plate), determined switch mechanism, hygromycin phenotype, *VSG427-2(221)* genotype, hygromycin genotype, and pseudogene genotype. A key for the abbreviations used is presented at the bottom of the table.
(PDF)

Table S2 **Switch type determination of** ***TERT^−/−^*** **short telomere clones.** Table presents the analysis of the 188 *TERT^−/−^* Short Telomere single-cell-isolated *VSG* switched secondary clones (supporting data for FIG. 4C – Green Bars). The table columns from left to right are: isolate identifier (codified name based on originating 96-well plate), determined switch mechanism, hygromycin phenotype, *VSG427-2(221)* genotype, hygromycin genotype, and pseudogene genotype. A key for the abbreviations used is presented at the bottom of the table.
(PDF)

Table S3 **Additional analyses of switch type determination for wild-type and** ***TERT^−/−^*** **switched secondary clones.** **PART I: Complied** ***VSG*** **Switched Isolate DATA.** Contains 3 tables and a key of abbreviations used. The table entitled “Switch Mechanism Determination” presents the matrix of genotypes and phenotypes used to categorize the mechanism of switching. All the data from “WT Telomere” and “Short Telomere” secondary switched clones are compiled under the heading “Data Summary” in tables that show the “Number” of switchers in each category and the “Percent” of the population they represent. **PART II: Analysis of switch mechanism by clone population.** WT and Short Telomere data sets are presented with respect to their single-cell-cloned starting populations. Columns from left to right are: name of the parental population, measured OSF of the population, number of secondary switched clones within the population, each of the possible switch types and the proportion (%) of each type in that population (supporting data for FIG. 4B & C and FIG. S2). **PART III: Analysis of switch mechanism by isolation day.** WT and Short Telomere switchers are presented with respect to the day they were isolated post-MACS depletion and single-cell-cloning (supporting data for FIG. S3). **PART IV: Mathematical analysis demonstrating that the increase in Short Telomere switching can be accounted for by the increase in GC.** The value “F” is defined and shown for each possible type of switch for both WT and Short telomere secondary switched clones, from which ΣF is determined. The supporting mathematical formulas and resulting values are shown (supporting data to manuscript results section 4). **PART V: Independent switch isolate analysis.** Matrices of WT or Short Telomere starting populations against their known phenotypic and genotypic outcomes were used to derive the minimum possible number of switchers within each population. The sum of the number of switch types within each population produces the minimum number of switches that comprise the 189 WT and 188 Short Telomere secondary switch clones (supporting data to manuscript results section 4).
(PDF)
