# Supplementary figures and images for: Telomere Length Affects the Frequency and Mechanism of Antigenic Variation in Trypanosoma brucei
Source: PLoS Pathog. 2012 Aug 30;8(8):e1002900. doi: 10.1371/journal.ppat.1002900 (PMC3431348; doi:10.1371/journal.ppat.1002900)

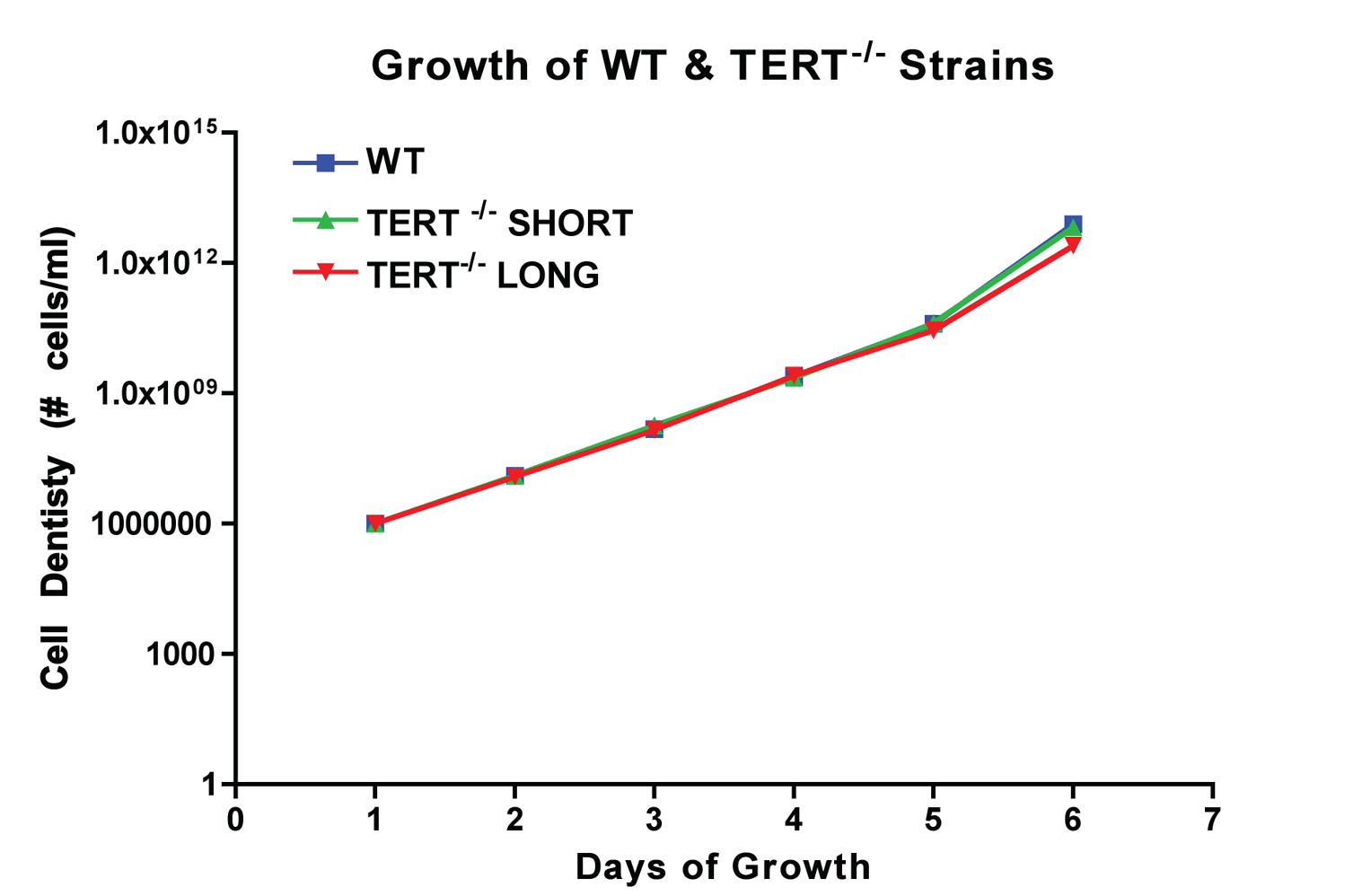

Supplement: Figure S1 — Growth of wild-type and telomerase mutant strains. Comparison of the growth of wild-type (WT) (blue) with TERT−/− short-telomere (green), and TERT−/− long-telomere (red) clones. (JPG) [file ppat.1002900.s001.jpg]

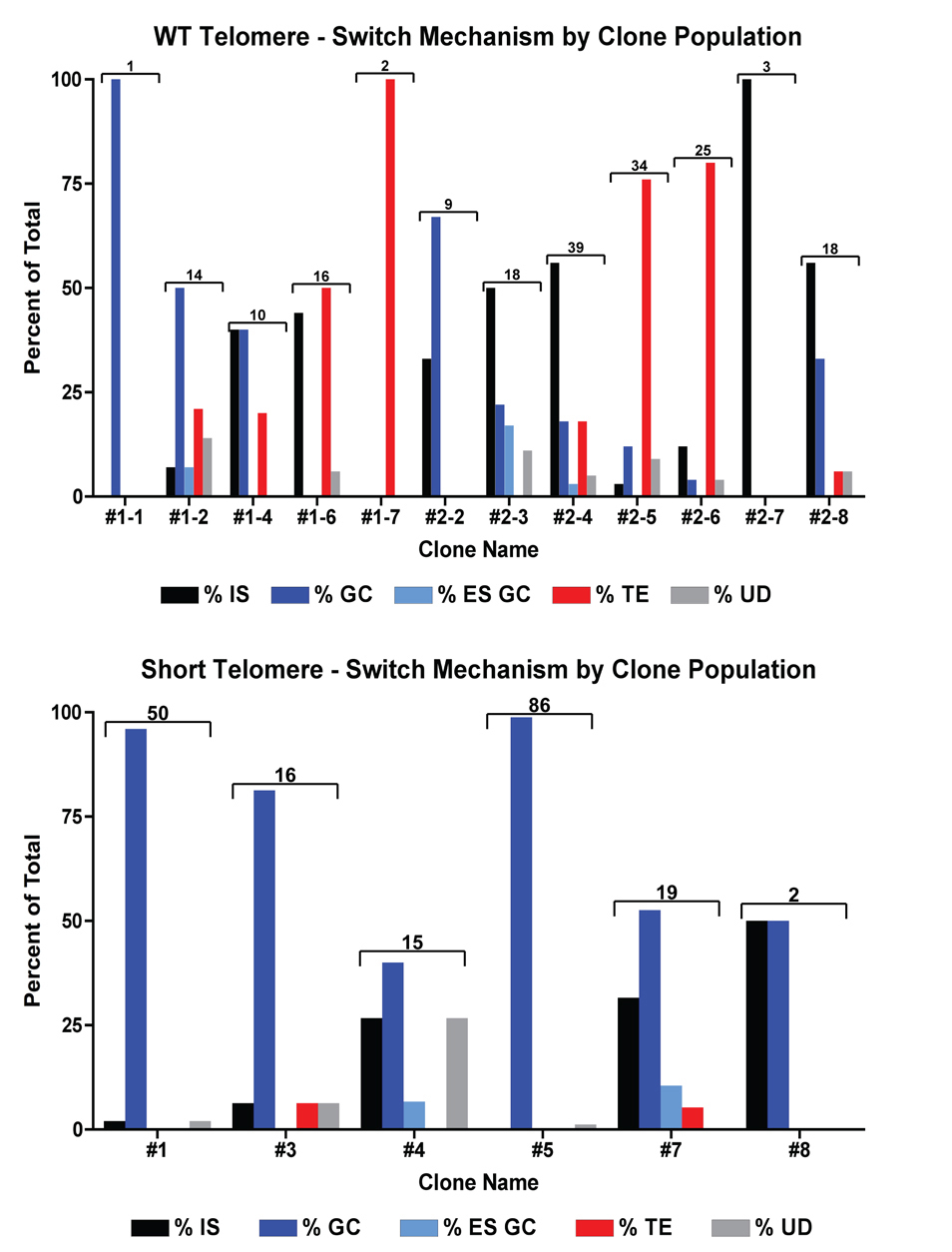

Supplement: Figure S2 — Mechanism of switching by clone population. Comparison of percent switch mechanism (IS: in situ [black], GC: gene conversion [dark blue], ES GC: expression site gene conversion [light blue], TE: telomere exchange [red], UD: undetermined [grey]) by starting clone population. Number of secondary switched clones in each population is shown above the bracket. Wild-type (WT) telomere populations are shown in top graph % short-telomere populations shown in bottom graph. (JPG) [file ppat.1002900.s002.jpg]

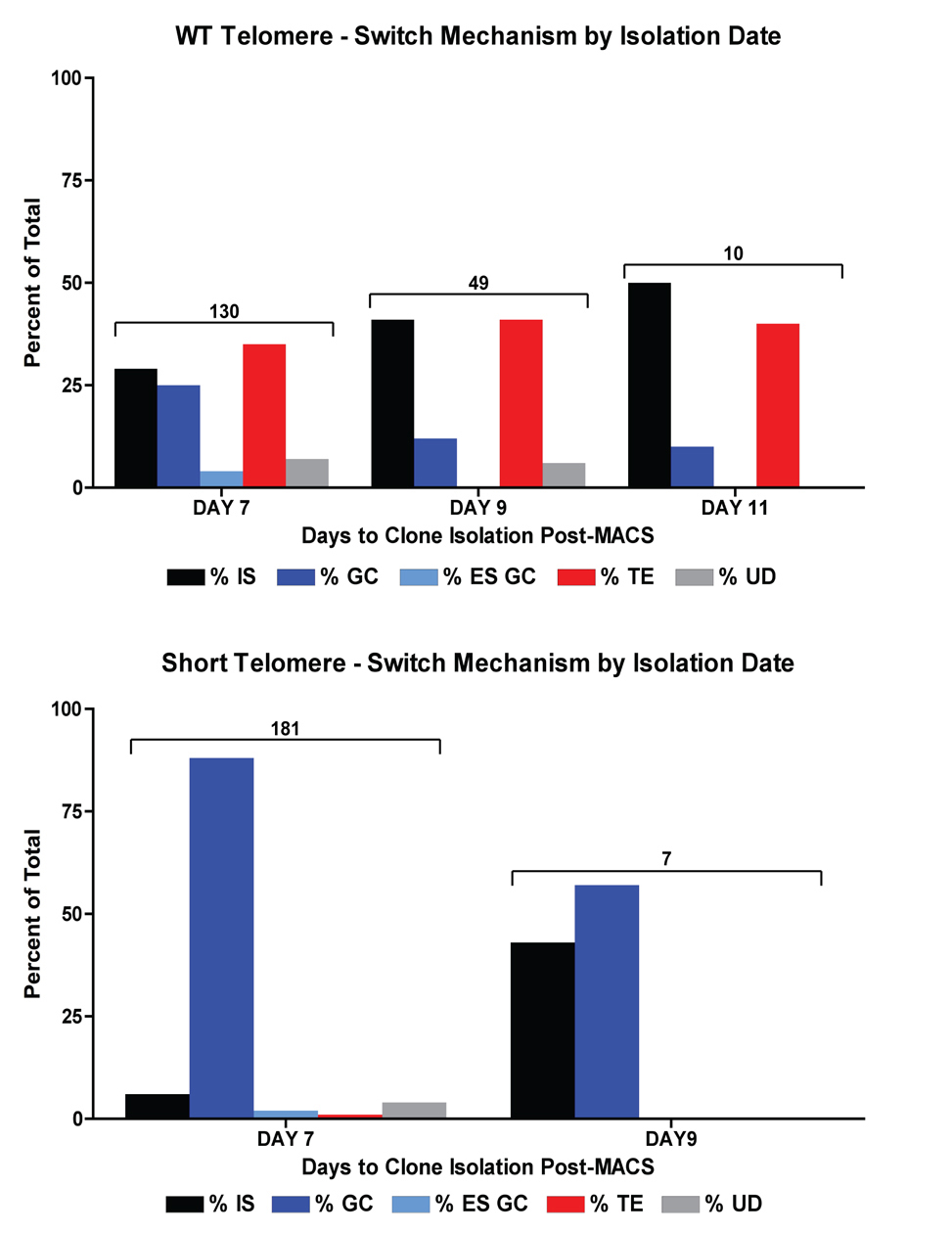

Supplement: Figure S3 — Mechanism of switching by clone isolation date. Comparison of percent switch mechanism (IS: in situ [black], GC: gene conversion [dark blue], ES GC: expression site gene conversion [light blue], TE: telomere exchange [red], UD: undetermined [grey]) by secondary clone isolation day. Number of secondary switched clones in each population is shown above the bracket. Wild-type (WT) telomere populations are shown in top graph and short telomere populations shown in bottom graph. (JPG) [file ppat.1002900.s003.jpg]
