# Supplementary material for: Telomere Length Affects the Frequency and Mechanism of Antigenic Variation in Trypanosoma brucei
Source: PLoS Pathog. 2012 Aug 30;8(8):e1002900. doi: 10.1371/journal.ppat.1002900 (PMC3431348; doi:10.1371/journal.ppat.1002900)
Supplement: Table S1 — Switch type determination of WT clones. Table presents the analysis of the 189 WT single-cell-isolated VSG switched secondary clones (supporting data for FIG. 4C – Blue Bars). The table columns from left to right are: isolate identifier (codified name based on originating 96-well plate), determined switch mechanism, hygromycin phenotype, VSG427-2(221) genotype, hygromycin genotype, and pseudogene genotype. A key for the abbreviations used is presented at the bottom of the table. (PDF) [file ppat.1002900.s004.pdf]

WT Telomere Switchers	Switch Mechanism	HYG (R/S)	221 (+/-)	HYG (+/-)	PSD (+/-)
**1-1B-A12**	GC	R	-	+	-
**1-2A-A1**	GC	R	-	+	+
**1-2A-A10**	GC	R	-	+	+
**1-2A-A12**	GC	R	-	+	-
**1-2A-D6**	UD	S	-	+	ND
**1-2A-E2**	GC	R	-	+	+
**1-2A-E9**	UD	S	-	+	ND
**1-2A-F11**	TE	R	+	+	ND
**1-2A-G2**	GC	R	-	+	+
**1-2A-G7**	IS	S	+	+	ND
**1-2A-H1**	TE	R	+	+	ND
**1-2A-H8**	GC	R	-	+	+
**1-2B-A11**	GC	R	-	+	+
**1-2B-B8**	ES GC	S	-	-	-
**1-2B-D1**	TE	R	+	+	ND
**1-4B-B1**	GC	R	-	+	-
**1-4B-D10**	GC	R	-	+	-
**1-4B-E2**	GC	R	-	+	-
**1-4B-E7**	IS	S	+	+	ND
**1-4B-H10**	TE	R	+	+	ND
**1-4C-A11**	TE	R	+	+	ND
**1-4C-A2**	IS	S	+	+	ND
**1-4C-D1**	GC	R	-	+	+
**1-4C-F6**	IS	S	+	+	ND
**1-4C-H9**	IS	S	+	+	ND
**1-6B-B5**	TE	R	+	+	ND
**1-6B-B7**	TE	R	+	+	ND
**1-6B-D1**	IS	S	+	+	ND
**1-6B-D11**	TE	R	+	+	ND
**1-6B-E1**	IS	S	+	+	ND
**1-6B-E7**	IS	S	+	+	ND
**1-6B-F1**	TE	R	+	+	ND
**1-6B-G6**	TE	R	+	+	ND
**1-6C-A10**	TE	R	+	+	ND
**1-6C-A5**	IS	S	+	+	ND
**1-6C-A6**	IS	S	+	+	ND
**1-6C-B11**	IS	S	+	+	ND
**1-6C-B4**	IS	S	+	+	ND
**1-6C-D3**	UD	S	-	+	ND
**1-6C-D5**	TE	R	+	+	ND
**1-6C-E11**	TE	R	+	+	ND

**1-7A-E1**	TE	R	+	+	ND
**1-7B-C5**	TE	R	+	+	ND
**2-2B-B5**	GC	R	-	+	-
**2-2B-E11**	IS	S	+	+	ND
**2-2B-H9**	IS	S	+	+	ND
**2-2C-C1**	IS	S	+	+	ND
**2-2C-C3**	GC	R	-	+	+
**2-2C-D10**	GC	R	-	+	-
**2-2C-E6**	GC	R	-	+	-
**2-2C-H10**	GC	R	-	+	-
**2-2C-H4**	GC	R	-	+	-
**2-3B-A10**	GC	R	-	+	-
**2-3B-A4**	GC	R	-	+	-
**2-3B-A6**	IS	S	+	+	ND
**2-3B-C1**	IS	S	+	+	ND
**2-3B-C6**	ES GC	S	-	-	+
**2-3B-D1**	IS	S	+	+	ND
**2-3B-D6**	IS	S	+	+	ND
**2-3B-E2**	IS	S	+	+	ND
**2-3B-E7**	IS	S	+	+	ND
**2-3B-F3**	ES GC	S	-	-	-
**2-3B-G4**	UD	S	-	+	ND
**2-3B-H4**	GC	R	-	+	-
**2-3C-A11**	UD	S	+	-	ND
**2-3C-C7**	ES GC	S	-	-	-
**2-3C-E7**	IS	S	+	+	ND
**2-3C-G7**	IS	S	+	+	ND
**2-3C-G9**	GC	R	-	+	-
**2-3C-H1**	IS	S	+	+	ND
**2-4A-A1**	IS	S	+	+	ND
**2-4A-A2**	IS	S	+	+	ND
**2-4A-A5**	GC	R	-	+	+
**2-4A-A6**	TE	R	+	+	ND
**2-4A-A8**	TE	R	+	+	ND
**2-4A-B4**	IS	S	+	+	ND
**2-4A-B6**	IS	S	+	+	ND
**2-4A-B7**	ES GC	S	-	-	+
**2-4A-C11**	GC	R	-	+	+
**2-4A-C3**	IS	S	+	+	ND
**2-4A-D10**	IS	S	+	+	ND
**2-4A-D6**	TE	R	+	+	ND
**2-4A-D9**	IS	S	+	+	ND
**2-4A-E10**	TE	R	+	+	ND

**2-4A-E2**	IS	S	+	+	ND
**2-4A-E6**	IS	S	+	+	ND
**2-4A-E9**	GC	R	-	+	+
**2-4A-F1**	IS	S	+	+	ND
**2-4A-F11**	GC	R	-	+	+
**2-4A-F5**	GC	R	-	+	+
**2-4A-F6**	IS	S	+	+	ND
**2-4A-G12**	TE	R	+	+	ND
**2-4A-G4**	GC	R	-	+	+
**2-4A-H11**	IS	S	+	+	ND
**2-4A-H2**	GC	R	-	+	+
**2-4A-H6**	IS	S	+	+	ND
**2-4B-A8**	IS	S	+	+	ND
**2-4B-B2**	UD	S	-	+	ND
**2-4B-C9**	IS	S	+	+	ND
**2-4C-A12**	UD	S	-	+	ND
**2-4C-B4**	TE	R	+	+	ND
**2-4C-C5**	IS	S	+	+	ND
**2-4C-D12**	IS	S	+	+	ND
**2-4C-D4**	IS	S	+	+	ND
**2-4C-F2**	TE	R	+	+	ND
**2-4C-G11**	IS	S	+	+	ND
**2-4C-G3**	IS	S	+	+	ND
**2-4C-H2**	IS	S	+	+	ND
**2-4C-H8**	IS	S	+	+	ND
**2-5B-A12**	TE	R	+	+	ND
**2-5B-A3**	GC	R	-	+	+
**2-5B-A5**	UD	S	-	+	ND
**2-5B-A6**	TE	R	+	+	ND
**2-5B-B12**	TE	R	+	+	ND
**2-5B-B5**	IS	S	+	+	ND
**2-5B-C10**	GC	R	-	+	-
**2-5B-C2**	TE	R	+	+	ND
**2-5B-D10**	TE	R	+	+	ND
**2-5B-D12**	TE	R	+	+	ND
**2-5B-D7**	TE	R	+	+	ND
**2-5B-E2**	TE	R	+	+	ND
**2-5B-E7**	TE	R	+	+	ND
**2-5B-E8**	UD	R	+	-	ND
**2-5B-F1**	TE	R	+	+	ND
**2-5B-F9**	TE	R	+	+	ND
**2-5B-G3**	TE	R	+	+	ND
**2-5B-G5**	TE	R	+	+	ND

**2-5B-H4**	TE	R	+	+	ND
**2-5C-A10**	TE	R	+	+	ND
**2-5C-A4**	TE	R	+	+	ND
**2-5C-B4**	TE	R	+	+	ND
**2-5C-C5**	TE	R	+	+	ND
**2-5C-C6**	TE	R	+	+	ND
**2-5C-D10**	UD	S	-	+	ND
**2-5C-E1**	TE	R	+	+	ND
**2-5C-E11**	TE	R	+	+	ND
**2-5C-F2**	GC	R	-	+	+
**2-5C-F5**	TE	R	+	+	ND
**2-5C-G1**	TE	R	+	+	ND
**2-5C-G5**	TE	R	+	+	ND
**2-5C-H2**	TE	R	+	+	ND
**2-5C-H5**	TE	R	+	+	ND
**2-5C-H9**	GC	R	-	+	-
**2-6A-A4**	TE	R	+	+	ND
**2-6A-A5**	TE	R	+	+	ND
**2-6A-B1**	TE	R	+	+	ND
**2-6A-B3**	IS	S	+	+	ND
**2-6A-C11**	TE	R	+	+	ND
**2-6A-D4**	TE	R	+	+	ND
**2-6A-D5**	TE	R	+	+	ND
**2-6A-D6**	TE	R	+	+	ND
**2-6A-E4**	GC	R	-	+	-
**2-6A-F5**	TE	R	+	+	ND
**2-6A-F9**	TE	R	+	+	ND
**2-6A-H3**	TE	R	+	+	ND
**2-6A-H6**	TE	R	+	+	ND
**2-6A-H8**	TE	R	+	+	ND
**2-6B-B1**	TE	R	+	+	ND
**2-6B-B10**	TE	R	+	+	ND
**2-6B-C2**	TE	R	+	+	ND
**2-6B-C6**	TE	R	+	+	ND
**2-6B-D9**	TE	R	+	+	ND
**2-6B-F12**	TE	R	+	+	ND
**2-6B-F7**	UD	S	-	+	ND
**2-6B-G1**	IS	S	+	+	ND
**2-6B-G10**	IS	S	+	+	ND
**2-6B-H10**	TE	R	+	+	ND
**2-6C-C4**	TE	R	+	+	ND
**2-7B-E5**	IS	S	+	+	ND
**2-7B-F9**	IS	S	+	+	ND

**2-7C-B10**	IS	S	+	+	ND
**2-8A-B9**	GC	R	-	+	-
**2-8A-D9**	GC	R	-	+	-
**2-8A-E11**	UD	S	+	-	ND
**2-8A-E7**	IS	S	+	+	ND
**2-8A-F12**	IS	S	+	+	ND
**2-8A-G2**	IS	S	+	+	ND
**2-8A-G7**	GC	R	-	+	ND
**2-8B-A8**	IS	S	+	+	ND
**2-8B-B1**	IS	S	+	+	ND
**2-8B-E9**	TE	R	+	+	ND
**2-8B-G3**	GC	R	-	+	-
**2-8C-A1**	GC	R	-	+	-
**2-8C-A3**	GC	R	-	+	-
**2-8C-C3**	IS	S	+	+	ND
**2-8C-D1**	IS	S	+	+	ND
**2-8C-F2**	IS	S	+	+	ND
**2-8C-F9**	IS	S	+	+	ND
**2-8C-H9**	IS	S	+	+	ND

KEY

**221=**	**VSG427-2**
**HYG=**	**Hygromycin**
**PSD=**	**Pseudogene**
**R=**	**Resistant**
**S=**	**Sensitive**
**+=**	**Present in Genome**
**-=**	**Absent from Genome**
**GC=**	**Duplicative Gene Conversion**
**IS=**	**In Situ/Transcriptional**
**TE=**	**Telomere Exchange**
**ES GC=**	**Expression Site GC**
**UD=**	**Undetermined**
