# Supplementary material for: Telomere Length Affects the Frequency and Mechanism of Antigenic Variation in Trypanosoma brucei
Source: PLoS Pathog. 2012 Aug 30;8(8):e1002900. doi: 10.1371/journal.ppat.1002900 (PMC3431348; doi:10.1371/journal.ppat.1002900)
Supplement: Table S2 — Switch type determination of TERT−/− short telomere clones. Table presents the analysis of the 188 TERT−/− Short Telomere single-cell-isolated VSG switched secondary clones (supporting data for FIG. 4C – Green Bars). The table columns from left to right are: isolate identifier (codified name based on originating 96-well plate), determined switch mechanism, hygromycin phenotype, VSG427-2(221) genotype, hygromycin genotype, and pseudogene genotype. A key for the abbreviations used is presented at the bottom of the table. (PDF) [file ppat.1002900.s005.pdf]

Short Telomere Switchers	Switch Mechanism	BSD (S/R)	221 (+/-)	BSD (+/-)	PSD (+/-)
**1A-A5**	GC	R	-	+	+
**1A-A7**	UD	S	-	+	ND
**1A-A8**	GC	R	-	+	-
**1A-A9**	GC	R	-	+	-
**1A-B10**	GC	R	-	+	-
**1A-B11**	GC	R	-	+	-
**1A-B3**	GC	R	-	+	-
**1A-B4**	GC	R	-	+	+
**1A-B5**	GC	R	-	+	-
**1A-B7**	IS	S	+	+	ND
**1A-C11**	GC	R	-	+	-
**1A-C12**	GC	R	-	+	-
**1A-C2**	GC	R	-	+	-
**1A-C4**	GC	R	-	+	-
**1A-D11**	GC	R	-	+	-
**1A-D3**	GC	R	-	+	-
**1A-D4**	GC	R	-	+	-
**1A-D5**	GC	R	-	+	-
**1A-D6**	GC	R	-	+	-
**1A-D7**	GC	R	-	+	+
**1A-D8**	GC	R	-	+	-
**1A-E12**	GC	R	-	+	+
**1A-E2**	GC	R	-	+	-
**1A-E3**	GC	R	-	+	-
**1A-E7**	GC	R	-	+	-
**1A-F10**	GC	R	-	+	-
**1A-F12**	GC	R	-	+	-
**1A-F3**	GC	R	-	+	+
**1A-F4**	GC	R	-	+	-
**1A-F5**	GC	R	-	+	-
**1A-F6**	GC	R	-	+	-
**1A-F7**	GC	R	-	+	-
**1A-F8**	GC	R	-	+	-
**1A-F9**	GC	R	-	+	-
**1A-G11**	GC	R	-	+	-
**1A-G2**	GC	R	-	+	-
**1A-G3**	GC	R	-	+	-
**1A-G5**	GC	R	-	+	-
**1A-G8**	GC	R	-	+	-
**1A-H10**	GC	R	-	+	-

**1A-H11**	GC	R	-	+	-
**1A-H3**	GC	R	-	+	-
**1A-H4**	GC	R	-	+	+
**1A-H5**	GC	R	-	+	-
**1A-H7**	GC	R	-	+	-
**1B-A3**	GC	R	-	+	-
**1B-A4**	GC	R	-	+	+
**1B-D9**	GC	R	-	+	-
**1B-E1**	GC	R	-	+	-
**1B-F3**	GC	R	-	+	-
**3A-A5**	TE	R	+	+	ND
**3A-A8**	GC	R	-	+	-
**3A-B8**	GC	R	-	+	-
**3A-C10**	GC	R	-	+	-
**3A-C11**	GC	R	-	+	-
**3A-D1**	IS	S	+	+	ND
**3A-D10**	GC	R	-	+	-
**3A-D2**	GC	R	-	+	-
**3A-D9**	GC	R	-	+	-
**3A-E11**	GC	R	-	+	-
**3A-E6**	GC	R	-	+	-
**3A-G6**	GC	R	-	+	-
**3B-F6**	GC	R	-	+	-
**3B-H3**	GC	R	-	+	-
**3B-H9**	GC	R	-	+	-
**3C-D2**	UD	S	-	+	ND
**4A-B10**	GC	R	-	+	-
**4A-B11**	ES GC	S	-	-	-
**4A-B6**	IS	S	+	+	ND
**4A-C9**	UD	S	-	+	ND
**4A-D7**	UD	S	-	+	ND
**4A-D8**	GC	R	-	+	-
**4A-E7**	GC	R	-	+	-
**4A-F10**	UD	S	-	+	ND
**4A-F8**	IS	S	+	+	ND
**4A-G12**	IS	S	+	+	ND
**4A-G3**	IS	S	+	+	ND
**4A-G5**	GC	R	-	+	+
**4A-G8**	GC	R	-	+	-
**4A-H8**	UD	S	-	+	ND
**4B-H5**	GC	R	-	+	+
**5A-A11**	GC	R	-	+	-
**5A-A12**	GC	R	-	+	-

**5A-A2**	GC	R	-	+	-
**5A-A4**	GC	R	-	+	-
**5A-A6**	GC	R	-	+	-
**5A-A7**	GC	R	-	+	-
**5A-A8**	GC	R	-	+	-
**5A-A9**	GC	R	-	+	-
**5A-B1**	GC	R	-	+	-
**5A-B10**	GC	R	-	+	-
**5A-B11**	GC	R	-	+	-
**5A-B12**	GC	R	-	+	-
**5A-B2**	GC	R	-	+	-
**5A-B4**	GC	R	-	+	-
**5A-B5**	GC	R	-	+	-
**5A-B7**	GC	R	-	+	-
**5A-B9**	GC	R	-	+	-
**5A-C1**	GC	R	-	+	-
**5A-C10**	GC	R	-	+	-
**5A-C11**	GC	R	-	+	-
**5A-C12**	GC	R	-	+	-
**5A-C2**	GC	R	-	+	-
**5A-C3**	GC	R	-	+	-
**5A-C6**	GC	R	-	+	-
**5A-C8**	GC	R	-	+	-
**5A-D1**	GC	R	-	+	-
**5A-D11**	GC	R	-	+	-
**5A-D12**	GC	R	-	+	-
**5A-D2**	GC	R	-	+	-
**5A-D4**	GC	R	-	+	-
**5A-D6**	GC	R	-	+	-
**5A-D8**	GC	R	-	+	-
**5A-D9**	GC	R	-	+	-
**5A-E1**	GC	R	-	+	-
**5A-E2**	GC	R	-	+	-
**5A-E3**	GC	R	-	+	-
**5A-E4**	GC	R	-	+	-
**5A-E5**	GC	R	-	+	-
**5A-E6**	GC	R	-	+	-
**5A-E7**	GC	R	-	+	-
**5A-E8**	GC	R	-	+	-
**5A-F1**	GC	R	-	+	-
**5A-F10**	GC	R	-	+	-
**5A-F11**	GC	R	-	+	-
**5A-F12**	GC	R	-	+	-

**5A-F2**	GC	R	-	+	-
**5A-F3**	GC	R	-	+	-
**5A-F4**	GC	R	-	+	-
**5A-F5**	GC	R	-	+	-
**5A-F6**	GC	R	-	+	-
**5A-F7**	GC	R	-	+	-
**5A-F8**	GC	R	-	+	-
**5A-F9**	GC	R	-	+	-
**5A-G1**	GC	R	-	+	-
**5A-G10**	GC	R	-	+	-
**5A-G11**	GC	R	-	+	-
**5A-G12**	GC	R	-	+	-
**5A-G2**	GC	R	-	+	-
**5A-G3**	GC	R	-	+	-
**5A-G4**	GC	R	-	+	-
**5A-G5**	GC	R	-	+	-
**5A-G6**	GC	R	-	+	-
**5A-G8**	GC	R	-	+	-
**5A-G9**	GC	R	-	+	-
**5A-H10**	GC	R	-	+	-
**5A-H2**	GC	R	-	+	-
**5A-H3**	GC	R	-	+	-
**5A-H5**	GC	R	-	+	-
**5A-H6**	GC	R	-	+	-
**5A-H7**	GC	R	-	+	-
**5A-H8**	GC	R	-	+	-
**5B-A2**	GC	R	-	+	-
**5B-A3**	GC	R	-	+	-
**5B-B7**	GC	R	-	+	-
**5B-C2**	GC	R	-	+	-
**5B-C6**	GC	R	-	+	-
**5B-D10**	GC	R	-	+	-
**5B-D6**	GC	R	-	+	-
**5B-E6**	GC	R	-	+	-
**5B-F6**	UD	S	-	+	ND
**5B-G5**	GC	R	-	+	-
**5B-H12**	GC	R	-	+	-
**5C-A5**	GC	R	-	+	-
**5C-F12**	GC	R	-	+	-
**5C-F4**	GC	R	-	+	-
**5C-G11**	GC	R	-	+	-
**7A-A12**	ES GC	S	-	-	-
**7A-A4**	IS	S	+	+	ND

**7A-A8**	IS	S	+	+	ND
**7A-B6**	IS	S	+	+	ND
**7A-B7**	GC	R	-	+	+
**7A-B9**	GC	R	-	+	+
**7A-D12**	TE	R	+	+	ND
**7A-D7**	GC	R	-	+	+
**7A-E12**	GC	R	-	+	+
**7A-E2**	ES GC	S	-	-	-
**7A-F10**	IS	S	+	+	ND
**7A-F12**	GC	R	-	+	+
**7A-F4**	GC	R	-	+	+
**7A-H11**	GC	R	-	+	+
**7A-H3**	GC	R	-	+	+
**7A-H5**	IS	S	+	+	ND
**7B-A7**	IS	S	+	+	ND
**7B-D7**	GC	R	-	+	-
**7B-H7**	GC	R	-	+	-
**8A-C10**	GC	R	-	+	-
**8A-D8**	IS	S	+	+	+

KEY

**221=**	**VSG427-2**
**BSD=**	**Blasticidin**
**PSD=**	**Pseudogene**
**R=**	**Resistant**
**S=**	**Sensitive**
**+=**	**Present in Genome**
**-=**	**Absent from Genome**
**GC=**	**Duplicative Gene Conversion**
**IS=**	**In Situ/Transcriptional**
**TE=**	**Telomere Exchange**
**ES GC=**	**Expression Site GC**
**UD=**	**Undetermined**
