# Supplementary material for: Telomere Length Affects the Frequency and Mechanism of Antigenic Variation in Trypanosoma brucei
Source: PLoS Pathog. 2012 Aug 30;8(8):e1002900. doi: 10.1371/journal.ppat.1002900 (PMC3431348; doi:10.1371/journal.ppat.1002900)
Supplement: Table S3 — Additional analyses of switch type determination for wild-type and TERT−/− switched secondary clones. PART I: Complied VSG Switched Isolate DATA. Contains 3 tables and a key of abbreviations used. The table entitled “Switch Mechanism Determination” presents the matrix of genotypes and phenotypes used to categorize the mechanism of switching. All the data from “WT Telomere” and “Short Telomere” secondary switched clones are compiled under the heading “Data Summary” in tables that show the “Number” of switchers in each category and the “Percent” of the population they represent. PART II: Analysis of switch mechanism by clone population. WT and Short Telomere data sets are presented with respect to their single-cell-cloned starting populations. Columns from left to right are: name of the parental population, measured OSF of the population, number of secondary switched clones within the population, each of the possible switch types and the proportion (%) of each type in that population (supporting data for FIG. 4B & C and FIG. S2). PART III: Analysis of switch mechanism by isolation day. WT and Short Telomere switchers are presented with respect to the day they were isolated post-MACS depletion and single-cell-cloning (supporting data for FIG. S3). PART IV: Mathematical analysis demonstrating that the increase in Short Telomere switching can be accounted for by the increase in GC. The value “F” is defined and shown for each possible type of switch for both WT and Short telomere secondary switched clones, from which ΣF is determined. The supporting mathematical formulas and resulting values are shown (supporting data to manuscript results section 4). PART V: Independent switch isolate analysis. Matrices of WT or Short Telomere starting populations against their known phenotypic and genotypic outcomes were used to derive the minimum possible number of switchers within each population. The sum of the number of switch types within each population produces the minimum number [file ppat.1002900.s006.pdf]

COMPILED *VSG* SWITCHED ISOLATE DATA

SWITCH MECHANISM DETERMINATION

Switch Type	Marker (R/S)	221 (+/-)	Marker (+/-)
**IS**	S	+	+
**GC**	R	-	+
**ES GC**	S	-	-
**TE**	R	+	+

KEY

OSF= Observed *VSG* Switching Frequency
PSD= Pseudogene
GC= Duplicative Gene Conversion
IS= In Situ/Transcriptional
TE= Telomere Exchange
ES GC= Expression Site GC
UD= Undetermined
221= VSG427-2

DATA SUMMARY

WT Telomere

NAME	NUMBER	PERCENT
**Switchers**	189	-
**IS**	63	33%
**GC**	40	21%
**ES GC**	5	3%
**TE**	69	37%
**UD**	12	6%
**GC Total**	45	24%
**GC+ PSD-**	26	58%
**AVG OSF**	1.38	-

Short Telomere

NAME	NUMBER	PERCENT
**Switchers**	188	-
**IS**	13	7%
**GC**	163	87%
**ES GC**	3	2%
**TE**	2	1%
**UD**	7	4%
**GC Total**	166	88%
**GC+ PSD-**	149	90%
**AVG OSF**	8.84	-

ANALYSIS OF SWITCH MECHANISM BY CLONE POPULATION

WT Telomere

Pop. Name	OSF (x10^-5)	# Switchers	IS	GC	ES GC	TE	UD	% IS	% GC	% ES GC	% TE	% UD
**#1-1**	0.84	1	0	1	0	0	0	0%	100%	0%	0%	0%
**#1-2**	0.81	14	1	7	1	3	2	7%	50%	7%	21%	14%
**#1-4**	1.06	10	4	4	0	2	0	40%	40%	0%	20%	0%
**#1-6**	2.72	16	7	0	0	8	1	44%	0%	0%	50%	6%
**#1-7**	0.51	2	0	0	0	2	0	0%	0%	0%	100%	0%
**#2-2**	2.10	9	3	6	0	0	0	33%	67%	0%	0%	0%
**#2-3**	1.76	18	9	4	3	0	2	50%	22%	17%	0%	11%
**#2-4**	1.87	39	22	7	1	7	2	56%	18%	3%	18%	5%
**#2-5**	1.63	34	1	4	0	26	3	3%	12%	0%	76%	9%
**#2-6**	1.27	25	3	1	0	20	1	12%	4%	0%	80%	4%
**#2-7**	1.06	3	3	0	0	0	0	100%	0%	0%	0%	0%
**#2-8**	0.98	18	10	6	0	1	1	56%	33%	0%	6%	6%
**TOTALS**		**189**	**63**	**40**	**5**	**69**	**12**					
**PERCENT**			**33.3%**	**21.2%**	**2.6%**	**36.5%**	**6.3%**					

Short Telomere

Pop. Name	OSF (x10^-5)	# Switchers	IS	GC	ES GC	TE	UD	% IS	% GC	% ES GC	% TE	% UD
**#1**	6.10	50	1	48	0	0	1	2%	96%	0%	0%	2%
**#3**	3.82	16	1	13	0	1	1	6%	81%	0%	6%	6%
**#4**	10.76	15	4	6	1	0	4	27%	40%	7%	0%	27%
**#5**	7.47	86	0	85	0	0	1	0%	99%	0%	0%	1%
**#7**	8.59	19	6	10	2	1	0	32%	53%	11%	5%	0%
**#8**	16.30	2	1	1	0	0	0	50%	50%	0%	0%	0%
**TOTALS**		**188**	**13**	**163**	**3**	**2**	**7**					
**PERCENT**			**6.9%**	**86.7%**	**1.6%**	**1.1%**	**3.7%**					

ANALYSIS OF SWITCH MECHANISM BY ISOLATION DAY
(Number of days post-MACs single cell cloning required for visible growth and isolation)

WT Telomere

Isolation Day	# Switchers	IS	GC	ES GC	TE	UD	% IS	% GC	% ES GC	% TE	% UD
**7**	130	38	33	5	45	9	29%	25%	4%	35%	7%
**9**	49	20	6	0	20	3	41%	12%	0%	41%	6%
**11**	10	5	1	0	4	0	50%	10%	0%	40%	0%

Short Telomere

Isolation Day	# Switchers	IS	GC	ES GC	TE	UD	% IS	% GC	% ES GC	% TE	% UD
**7**	181	10	159	3	2	7	6%	88%	2%	1%	4%
**9**	7	3	4	0	0	0	43%	57%	0%	0%	0%

GC ACCOUNTS FOR THE INCREASED OSF OF SHORT TELOMERE CLONES

If $\Sigma \mathbf{F}_{Short} \cong \Sigma \mathbf{F}_{WT} + \Delta \mathbf{F}_{GC}$, then GC accounts for the increase in Short telomere OSF

Where: **F**=Average OSF x % Mechanism & $\Delta \mathbf{F}_{GC} = \mathbf{F}_{GC,Short} - \mathbf{F}_{GC,WT}$

WT	F
$\mathbf{F}_{IS}$	0.46
$\mathbf{F}_{GC}$	0.33
$\mathbf{F}_{TE}$	0.50
$\mathbf{F}_{UD}$	0.09
$\Sigma \mathbf{F}_{WT}$	1.38

Short	F
$\mathbf{F}_{IS}$	0.61
$\mathbf{F}_{GC}$	7.81
$\mathbf{F}_{TE}$	0.09
$\mathbf{F}_{UD}$	0.33
$\Sigma \mathbf{F}_{Short}$	8.84

8.86

$\Delta \mathbf{F}_{GC} =$ 7.48 $\Sigma \mathbf{F}_{WT} + \Delta \mathbf{F}_{GC} =$ **8.86**, which is $\cong$ to $\Sigma \mathbf{F}_{Short}$

Therefore, the increase in OSF in short telomere clones can be accounted for by the percent increase in GC

INDEPENDENT SWITCH ISOLATE ANALYSIS

WT Telomere Pop.	Total	GC 224- PSD+	GC 224- PSD-	GC 224+ PSD+	GC 224+ PSD-	IS 224-	IS 224+	TE 224-	TE 224+	UD 224-	UD 224+	ESGC 224-	ESGC 224+	# switch types
**#1-1**	1	0	1	0	0	0	0	0	0	0	0	0	0	1
**#1-2**	14	6	1	0	0	1	0	3	0	2	0	1	0	6
**#1-4**	10	1	3	0	0	4	0	2	0	0	0	0	0	4
**#1-6**	16	0	0	0	0	7	1	8	0	0	0	0	0	3
**#1-7**	2	0	0	0	0	0	0	2	0	0	0	0	0	1
**#2-2**	9	1	5	0	0	2	1	0	0	0	0	0	0	4
**#2-3**	18	0	2	0	2	9	0	0	0	2	0	2	1	6
**#2-4**	39	0	0	7	0	21	1	7	0	2	0	1	0	6
**#2-5**	34	1	2	1	0	1	0	26	0	3	0	0	0	6
**#2-6**	25	0	1	0	0	0	3	20	0	1	0	0	0	4
**#2-7**	3	0	0	0	0	3	0	0	0	0	0	0	0	1
**#2-8**	18	0	0	1	5	10	0	1	0	1	0	0	0	5
	189													47

Short Telomere Pop.	Total	GC 224- PSD+	GC 224- PSD-	GC 224+ PSD+	GC 224+ PSD-	IS 224-	IS 224+	TE 224-	TE 224+	UD 224-	UD 224+	ESGC 224-	ESGC 224+	# switch types
**#1**	50	5	18	1	24	1	0	0	0	1	0	0	0	6
**#3**	16	0	13	0	0	1	0	1	0	1	0	0	0	4
**#4**	15	2	4	0	0	4	0	0	0	4	0	1	0	5
**#5**	86	0	82	0	3	0	0	0	0	1	0	0	0	3
**#7**	19	8	2	0	0	6	0	1	0	0	0	2	0	5
**#8**	2	0	1	0	0	1	0	0	0	0	0	0	0	2
	188													25

Highlighted wells count as a unique switch type within population
